# Effects of *Veratrilla baillonii* Extract on Hepatic Gene Expression Profiles in Response to *Aconitum brachypodum*-Induced Liver Toxicity in Mice

**DOI:** 10.3389/fphar.2019.00568

**Published:** 2019-05-31

**Authors:** Jun Li, Gang Liu, Awais Ihsan, Xuejia Yi, Da-Gui Wang, Han Cheng, Azhar Muhammad, Xian-Ju Huang

**Affiliations:** ^1^College of Pharmacy, South-Central University for Nationalities, Wuhan, China; ^2^Department of Pharmacy, Renmin Hospital of Wuhan University, Wuhan, China; ^3^Department of Biosciences, COMSATS University Islamabad, Sahiwal, Pakistan

**Keywords:** *Veratrilla baillonii* extract, hepatic gene expression, *Aconitum brachypodum*, liver toxicity, hepato-protective mechanism

## Abstract

This manuscript was aimed to explore the hepato-protective effect of water extract of *Veratrilla baillonii* Franch. (Gentianaceae) (WVBF) on serious hepatic toxicity induced in mice treated with *Aconitum brachypodum* Diels (Ranunculaceae) at transcriptome level. The physiological and pathological symptoms were evaluated as the markers for hepato toxicity induced by *A. brachypodum* Diels (CFA) extracted compounds. Moreover, gene chip method was used to compare and investigate the gene expression level of WVBF on CFA induced-liver toxicity to identify the potential target of WVBF and CFA on liver. The results showed that WVBF had a significant detoxification effect on CFA-induced acute hepatic toxicity. There were 130 genes with lower expression and 124 genes expressed at higher rate in CFA treated group as compared with normal control group, while there are 67 genes down-regulated and 74 genes up-regulated in WVBF treated group in comparison with CFA treated group. WVBF could attenuate CFA-induced liver damage in mice through regulating oxidative stress, inflammatory injury and cell apoptosis/necrosis pathways. On the other hand, WVBF and CFA may have potential synergetic effects on the target genes of certain diseases such as inflammation, cancer and diabetes.

## Introduction

**FIGURE 1:**
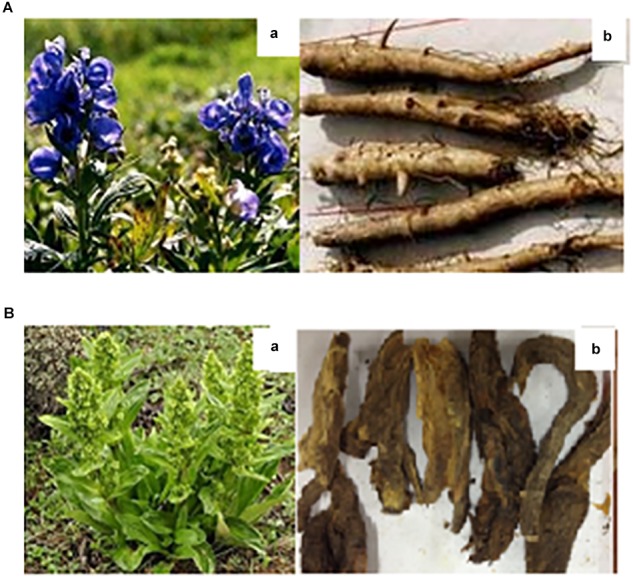
The morphology of *Aconitum brachypodum* and *Veratrilla baillonii* Franch. **(A-a)** Original plant of *A. brachypodum*, **(A-b)** the dehydrated roots of *A. brachypodum*. **(B-a)** Original plant of *V. baillonii* Franch, **(B-b)** the dehydrated roots of *V. baillonii* Franch.

*Aconitum brachypodum* Diels (Ranunculaceae) is a perennial herb, 50 ∼ 70 cm tall, leaves alternate, terminal raceme, violet, the root of the plant is used as traditional Chinese medicine (TCM) (Figure 1A) (Bisset, 1981). *A. brachypodum* extracts (aconite alkaloids) have been reported to exhibit several clinically important pharmacological activities such as curing pain, Anti-inflammatory, Antinociceptive, Analgesic, Anti-arrhythmic etc. (Herzog et al., 1964; Zhao et al., 2012; Zyuz’kov et al., 2012). Although *A. brachypodum* derivative compounds such as aconitine and related alkaloids which have been used to treat many diseases in various parts of china especially remote areas such as Tibet at domestic level (Liu et al., 2014; Ma et al., 2015). However, inadequate processing and excessive dosage can cause toxicity resulting in acute poisoning (Chan, 2009; Singhuber et al., 2009). After all, clinically relevant poisoning happen occasionally and there is not an effective way to rescue this poisoning caused by aconite compounds extracted from *Aconitum*.

*Veratrilla baillonii* Franch. (Gentianaceae) is a perennial herb, 30 ∼ 80 cm tall, cauline leaves oval, cone compound cyme, the root of the plant is generally used as a Chinese traditional medicine (Figure 1B). *V. baillonii* can be discovered in many parts of western China (Zyuz’kov et al., 2012). *V. baillonii* associated microsatellite markers and transcriptome analyses have been reported recently (Herzog et al., 1964). The discovery of these novel markers reveals genetic diversity in different regions of china such as, Jinsha River and Mekong River (Ma et al., 2015). *V. baillonii* Franch has been used as herbal medicine by Lisu and Naxi people from decades in Yunnan Region, with the effect of heat-clearing and detoxifying, which has been mainly used in the treatment of lung heat cough, amygdalitis, gastritis, diarrhea, chronic cholecystitis, burn, bruises and carbuncle sore swollen poison etc., especially for antitoxic on *Aconitum* plants (Herzog et al., 1964; Zyuz’kov et al., 2012; Liu et al., 2014). Hence TCM not only facilitate people at local level medically, but also bring benefits to clinic.

Our previous studies submitted that *A. brachypodum* (CFA) associated toxicity may effect various organs such as kidney, liver, heart, and CNS system. The water extract of *V. baillonii* (WVBF) could attenuate acute toxicity and pathological changes in mice induced by treatment of chloroform fraction of CFA to a certain extent (Ge et al., 2016; Yu et al., 2016). Another study revealed that *V. baillonii* extracted chemical compounds increased Akt phosphorylation to inhibit *Pck1* in liver cells, indicating its active control of blood glucose in animal models of diabetes (Huang et al., 2016). However, the deep molecular mechanism and the molecular targets remain unknown.

Since the hepato-protective mechanism of WVBF has not been investigated before, the current study aims to examine the pathogenic effect of *A. brachypodum* and the potential detoxification mechanism of *V. baillonii* on liver at gene expression level. In this paper, acute hepatic toxicity in mice was induced by CFA treatment, and gene chip assay was used to analyze gene expression profile in liver of CFA induced mice in the presence or absence of WVBF.

## Materials and Methods

### Extract Preparation

*Veratrilla baillonii* Franch (Gentianaceae) and *Aconitum brachypodum* Diel (Ranunculaceae) plants dehydrated stocks were brought from the city of Kunming, Yunnan region in China. These plants were validated by Dr. Liu Xinqiao, who is affiliated to the Pharmacy department in South-central university of nationalities, China. WVBF and CFA were prepared and analyzed in the laboratory as formerly described (Ge et al., 2016). Moreover, the voucher specimens (No. S20140710 and No. S20130825) were preserved in Herbarium of Pharmacy College, South-Central University of Nationalities, Wuhan, Hubei, China.

### Laboratory Animals and Administration

Male Kunming mice (18–22 g) were acquired from Hubei Animal Center (Wuhan, China). These mice were maintained in a room with 12-h dark/light rotation, room temperature (20–24°C), and feed well before experiments. Animals were fasted for 12 h before experiment with access to water only in order to avoid the impacts of food absorption on the efficacy of reagents used. Animal protocols were followed according to the National Institutes of Health (NIH) rules (NRC, 2004) and approved by the Ethical Committee in South-central university of nationalities (No. yxy20141008).

The dosage of CFA as well as WVBF were selected according to our previous experiment (Ge et al., 2016). Fifteen male mice were randomly and equally divided into three groups. Before oral administration, all mice were fasted for 12 h with water and *ad libitum*. The first group (control) received sterile water orally. Next was the group single-injection CFA (30 mg/kg), further this group was treated orally for 10 mL/kg disinfected water for a span of 3 and 30 min. The third group treated with CFA (30 mg/kg), later on mice were monitored orally with WVBF at dose of 50 mg/kg, individually, for a span of 3 and 30 min (to see in Table 1).

**Table 1:** The division of animal groups and administration.

Group	Number	Name	Abbreviation
Group 1	5	Control	C
Group 2	5	CFA (30 mg/kg)	C30
Group 3	5	CFA + WVBF (30 mg/kg + 50 mg/kg)	C30 + W50


*During the 4-h time of post administration, clinical and physiological parameters were considered. Finally, mice were killed, serum biochemistry examinations were done using their blood samples and organs were dismembered, and stored for further analysis*.

### Clinical Biochemistry

All mice blood samples were collected and immediately added in the refrigerated centrifuge for 3,000 r/min and 10 min, we chose the upper plasma for the detection of blood biochemical indexes. The biochemistry of serum was examined using Synchron Clinical System CX4 (Beckman Coulter, Brea, CA, United States) considering the company’s instructions (Beijing Leadman Biochemistry Technology Co., Ltd., Beijing, China). In serum contents of alanine aminotransferase (ALT), aspartate aminotransferase (AST) and alkaline phosphatase (ALP) were analyzed.

### RNA Extraction and Microarray Analysis

Liver samples (three each) from Control, CFA and WVBF (50 mg/kg) groups were selected for microarray analysis. Total RNA was isolated from liver samples weighing 1 g each sample using Trizol (Invitrogen Grand Island, NY, United States). RNA quality was determined by using a spectrophotometer, model NanoDrop ND1000 and was evaluated by the RIN (RNA Integrity Number) value of 7 by means of an Agilent 2100 Bioanalyzer. Procedures such as sample labeling, staining, hybridization, and signal reading were carried out at Shanghai biotechnology corporation using the mice genome 4 × 44K microarray (Agilent). The data obtained from experiments were analyzed using GeneSpring software in version 12.6 (Agilent). The differential expressed genes were filtered with fold change (FC > |2|) and *t*-test (*P* < 0.05), and a clustered hierarchically by using the cluster program. Further genes were functionally classified on the basis of Kyoto Encyclopedia of Genes and Genomes (KEGG) and gene ontology (GO) exploration were performed using the SBC analysis system (SAS^1^). On the other hand, the annotation data were obtained from GeneBank and KEGG^2^. All the data were uploaded as a series number GSE27021 in the Gene Expression Omnibus (GEO) at the National Center for Biotechnology Information (NCBI) website. In the experiment, each group was applied with three replicated biological experiments for obtaining the definitive expressive level, which was then normalized by GeneSpring software and finally screened for differential expressed genes by *T*-test.

### Quantitative Real-Time PCR

After affymetrix microarray analysis, the remained mRNA samples were used to verify gene expression changes using quantitative RT-PCR. Due to the result of microarray analysis, the gene levels of TNF receptor associated factor2 (TRAF2) and cyclin-dependent kinase inhibitor 1A (P21) were selected to testify in the liver sample. cDNA was prepared using 1 μg of total RNA by means of the PrimeScript^TM^ RT reagent Kit (TaKaRa) according to the company’s manual. Reactions were carried out in a 20 μL well system using an Real-Time PCR system (Applied Biosystems) under the following conditions: 30 s at 95°C trailed by 40 cycles of 5 s heating at 95°C, and elongation 40 s at 60°C (7). The primer sequences used for amplification of target genes are listed as below:

TRAF2: fw ATGGCTGTACCTGGAGCAGAA and rw GCTGCCTTCTATACCTTCTGA;
P21: fw CCTGGTGATGTCCGACCTGTT and rw CCCCTTAGAAGTCCGGCGAG;
GAPDH: fw TGTGTCCGTCGTGGATCTGA and rw TTGCTGTTGAAGTCGCAGGAG.

### Statistical Analysis

SPSS (18.5) was used for statistical analysis. All the results were accounted as mean ± S.E.M. Variation among the groups was analyzed using one-way (ANOVA) test, monitored by LSD’s tests. A minimum range of variance *P* < 0.05 was reflected significant.

## Results

### Clinical Observations

As revealed in Table 2, a single administration of CFA (30 mg/kg) shows acute poisoning effects causing symptoms such as hypoactivity, hyperventilation, retching, scratching of mouth, dribbling, diaphoresis, protopsis, diarrhea, writhing and even hyperspasmia. Some of these symptoms are reversible after 1–2 h. In CFA group, 1 of 5 mice were found dead after 12 min of administration. However, WVBF (10–50 mg/kg) can attenuate the acute toxicity induced by CFA (30 mg/kg) as it can be seen that the toxic symptoms are reduced and no death was observed after oral gavage, which fully demonstrated the detoxification influence of WVBF on CFA-induced severe toxicity.

**Table 2:** Clinical observation of WVBF on CFA-induced acute toxicity in mice.

Dosage (mg/kg)	Death number	Observed changes				
		Retching	Hyperspasmia	Scratching mouth	Dribbling	Proptosis
Control	0	No	No	No	No	No
C30	1	Reversible after 30 min(3)	Reversible after 30 min(2)	Reversible after 2 h(4)	Reversible after 1 h(4)	Reversible after 30 min(2)
C30 + W50	0	Reversible after 15 min(3)	No	Reversible after 1 h(2)	Reversible after 30 min(3)	Reversible after 15 min (1)


### Serum Chemistry of Mice After Treatment of CFA and WVBF

**FIGURE 2:**
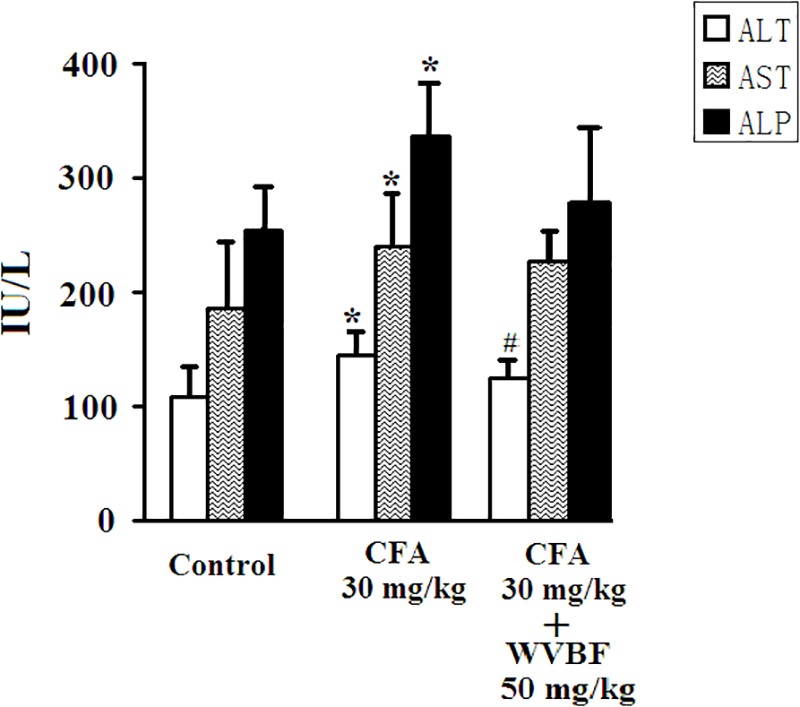
Serum biochemistry analyses of KM mice after CFA and WVBF administration. CFA accounts to the chloroform extraction of *A. brachypodum* Diel; WVBF accounts to the water decoction of *V. baillonii* Franch. Data presented as mean ± SD of 5 animals per group. ^∗^*P* < 0.05, significant difference vs. control group. ^#^*P* < 0.05, significant change vs. CFA group.

As presented in Figure 2, treated group assessed in comparison with control, the single treatment of CFA at 30 mg/kg led to significantly increase serum activities of ALT, AST, and ALP, demonstrating the liver injury induced by CFA (Ge et al., 2016; Huang et al., 2016; Yu et al., 2016). On the other hand, group of C + W50 decreased CFA-induced these parameters to some extend, especially led to a significant decrease of ALT, indicating that WVBF could attenuate the CFA-induced liver toxicity (Figure 2).

### The Effect of WVBF Treatment on CFA-Induced Changes of mRNA Expression Profiles in Mouse Liver

#### Analysis of Gene Expression Changes

**FIGURE 3:**
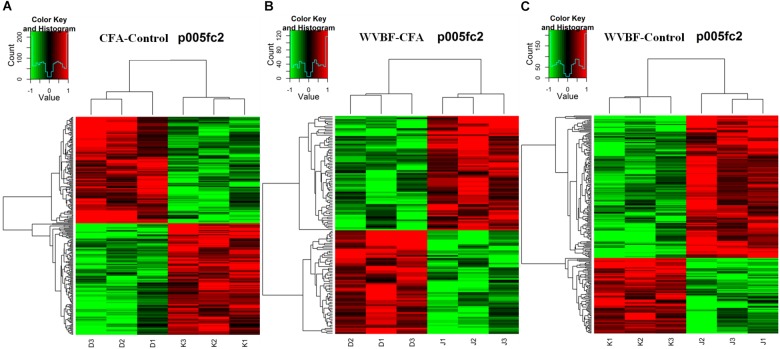
Heat map of the biased gene expression between **(A)** CFA/Control group, **(B)** WVBF/Control group, and **(C)** CFA/WVBF group. The heat map of the biased gene expression is identified by the hierarchical cluster analysis, and the gene expression is revealed in the heat map as up-regulated (red color), down-regulated (green color), and no change (black color).

The differences in expression of all genes in control, CFA, and C30 + W50 groups are displayed in volcano plot (Supplementary Figure S1) and scatter plot (Supplementary Figure S2), respectively. According to analysis expression levels of total 254 genes were altered in CFA group (Supplementary Figures S1, S2) as compared with normal control group, among which 130 were down-regulated and 124 are up-regulated. Supplementary Figures S1, S2 showed that WVBF treatment alters 231 gene expression as compared with normal control group, where 80 genes are down-regulated and 151 genes are up-regulated. By comparing Supplementary Figures S1, S2, we have observed that WVBF treatment alters 141 gene expression compared with CFA group, among which 67 genes are under expressed and 74 genes are highly expressed (*P* < 0.05 and Fold Change ≥ 2.0). A global view of the gene modulation in the livers of mice is shown in Figure 3.

Gene chip analysis revealed differentially expressed genes in three groups (A) CFA/Control group, (B) WVBF/Control (C) CFA/WVBF group. The heat map of the genes expressed differentially is identified by the hierarchical cluster analysis, and the gene expression is presented in the heat map as highly expressed (red color), low expressed (green color) and no change (black color). Heat map presents quantitative analysis of genes expressed in three main groups (A) CFA/Control group, (B) WVBF/Control (C) CFA/WVBF group. When we compare (A) CFA vs. control/group and (B) WVBF/control, higher number of genes are upregulated and lower number of genes are downregulated in CFA/control group as compared to WVBF. Whereas CFA/WVBF group represents lower number of upregulated and downregulated genes.

Hierarchical cluster analysis revealed that differential expression profiles of genes extracted from these samples were roughly classified, respectively. The significant differential expressions of genes were shown in Figure 3A–C. From the heat maps, significant changes in gene expression profiles of Control, CFA and CFA + WVBF groups could be observed, which indicated that effect of WVBF on CFA-treated mice might be through the regulation of gene expression profiles.

**FIGURE 4:**
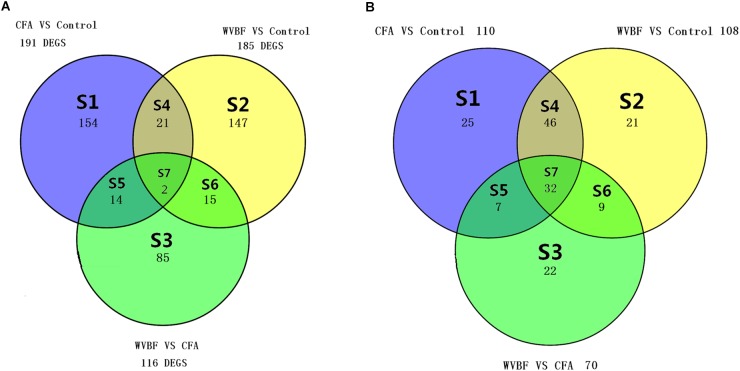
**(A)** Venn diagram displays the genes number in liver, which differs meaningfully (*P* < 0.05 and absolute fold change ≥ 2) between WVBF, CFA, and Control groups. **(B)** Venn diagram displays the number of shared pathways in liver of WVBF, CFA, and Control groups. The colors indicate the number of expressively modulated genes and pathways among Control, CFA, and WVBF groups in Figure 6.

Different sets (S1–S7) show the number of modulated genes among the three different groups in Figure 4A and pathways in Figure 4B, respectively. The changes in the Venn diagram also confirm the effect of WVBF on CFA-treated mice via regulation of gene expression profiles as described above.

#### GO Analysis

**FIGURE 5:**
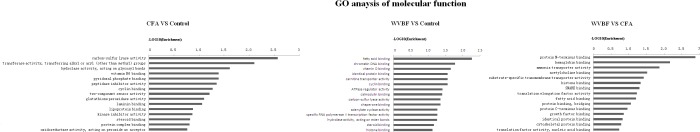
Gene ontology (GO) classification based on molecular function considering the differential gene expression using parameters, *P*-value < 0.05 and FDR < 0.05 to filter explicit results.

**FIGURE 6:**
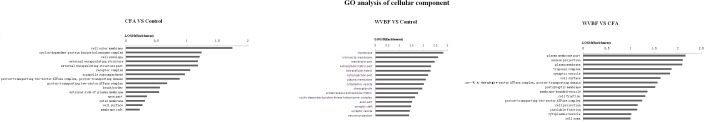
Gene ontology classification based on cellular component considering the differential gene expression using parameters, *P*-value < 0.05 and FDR < 0.05 to filter explicit results.

**FIGURE 7:**
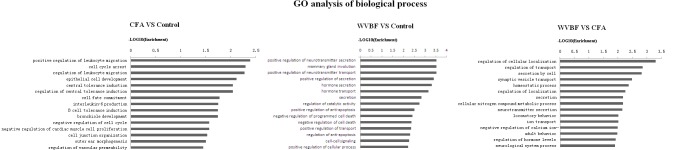
Gene ontology classification based on biological process considering the differential gene expression using parameters, *P*-value < 0.05 and FDR < 0.05 to filter explicit results.

To further explore the relationship between gene functions, GO-network among the C30 + W50, CFA and Control groups was constructed. GO analysis encompassed three domains: molecular function, biological process, and cellular component. The significant GO categories were selected with a P value less than 0.05 and fold change of 2 or higher. Moreover, through the GO analyses is graphic, significantly differentially expressed genes could be observed among the WVBF, CFA and control groups. All significant GO terms were summarized in Figure 5–7.

Figure 5 shows that in comparison with control group, the molecular function of genes that were differentially regulated by CFA mainly focused on carbon-sulfur lyase activity, transferase activity, hydrolase activity, pyridoxal phosphate binding, peptidase inhibitor activity and cyclin binding. While as for WVBF, its function mainly included regulation of fatty acid binding, chromatin DNA binding, vitamin D binding, cyclin binding and ATPase regulator activity. The interaction between WVBF and CFA included protein N-terminus binding, hemoglobin binding, ammonia transporter activity, acetylcholine binding and transmembrane transporter activity.

In respect of cellular component, CFA could affect cell outer membrane, cyclin-dependent protein kinase holoenzyme complex, cell envelope, while WVBF mainly influenced intrinsic membrane, membrane part and extracellular matrix. The interaction between WVBF and CFA included plasma membrane part, neuron projection, plasma membrane and troponin complex (Figure 7).

**FIGURE 8:**
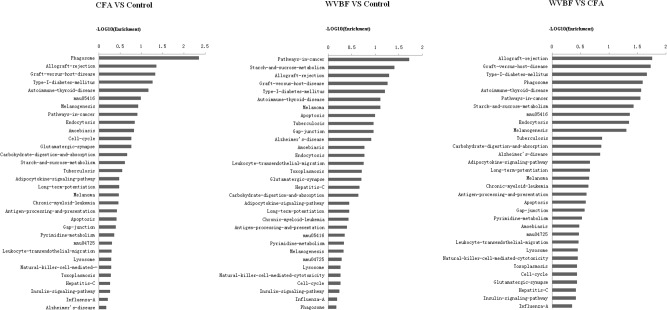
Pathway analysis of differentially expressed genes. *P* < 0.05 and FDR < 0.05 using parameters to filter explicit KEGG pathways. The vertical axis is the pathway category, and the horizontal represents the difference degree in the same pathway among WVBF, CFA, and control groups. Axis is the –log10 (Enrichment) of these significant pathways.

Analysis of biological process suggested that CFA may influence leukocyte migration, cell cycle arrest, leukocyte migration, epithelial cell development while WVBF could regulate secretion and transport of neurotransmitter, mammary gland involution, hormone secretion and transport. The interaction between WVBF and CFA included regulation of cellular localization, transport, secretion by cell, synaptic vesicle transport and homeostatic process (Figure 8).

Highly upregulated and downregulated (top 10) genes expression has been compared in three samples in CFA, WVBF, CFA vs. WVBF (Tables 3, 4). GO (MGI) analysis for these topmost regulated genes in CFA involved in cellular localization and proliferations, cell death and response to stimulus, whereas exceedingly expressed genes in WVBF and WVBF vs. CFA samples applies a protective effect on normal and CFA induced toxicity samples, respectively, by regulating biological pathways such as immune response, response to stimulus and to maintain the homeostatic condition (Supplementary Tables S1, S2).

**Table 3:** Top10 upregulated genes in CFA, WVBF, and WVBF vs. CFA samples.

Gene symbol	Genbank accession	GeneName	CFA vs. normal	WVBF vs. normal	WVBF vs. CFA
Zfp945	NM_177358	Zinc-finger-protein-945	16	29.5	NA
Itga2	NM_008396	Integrin-alpha-2	8.1	NA	NA
Cml5	NM_023493	Camello-like-5	7	NA	NA
4930579F01Rik	NM_001163385	RIKEN-cDNA-4930579F01-gene	5.6	NA	NA
Cspg5	NM_013884	Chondroitin-sulfate-proteoglycan-5	5	NA	NA
Ren1	NM_031192	Renin-1-structural	5	7.8	NA
Ren2	NM_031193	Renin-2-tandem-duplication-of-Ren1	4.7	8	NA
Ren1	NM_031192	Renin-1-structural	4.6	6.2	NA
Gadd45b	NM_008655	Growth-arrest-and-DNA-damage-inducible-45-beta	4.6	NA	NA
Wfdc12	NM_138684	WAP-four-disulfide-core-domain-12	4.6	NA	NA
Cabyr	NM_027687	Calcium-binding tyrosine-(Y)-phosphorylation regulated (fibrousheathin 2)	6.7	NA	NA
Adcy1	NM_009622	Adenylate cyclase 1	29.1	NA	NA
H2-Q1	NM_010390	Histocompatibility 2, Q region locus 1	12.7	NA	14.8
Ptgfr	NM_008966	Prostaglandin F receptor	10.8	NA	NA
Pnpla3	NM_054088	Patatin-like phospholipase domain containing 3	6.3	NA	NA
4930579F01Rik	NM_001163385	RIKEN cDNA 4930579F01 gene	6.1	NA	NA
Arhgap20os	NR_033560	Rho GTPase activating protein 20, opposite strand	NA	NA	6.1
Hyls1	NM_029762	Hydrolethalus syndrome 1	NA	NA	5.6
Chrna4	NM_015730	Cholinergic receptor, nicotinic, alpha polypeptide 4	NA	NA	5.3
Vgll3	NM_028572	Vestigial like 3 (Drosophila)	NA	NA	5
Krt2	NM_010668	Keratin 2	NA	NA	4.8
A430089I19Rik	NM_177913	RIKEN cDNA A430089I19 gene	NA	NA	4.7
Atp6v0d2	NM_175406	ATPase, H+ transporting, lysosomal V0 subunit D2	NA	NA	5
NA	BB505010	NA	NA	NA	5.9
Gabbr2	NM_001081141	Gamma-aminobutyric acid (GABA) B receptor, 2	NA	NA	7.2


**Table 4:** Top10 downregulated genes in CFA, WVBF, and WVBF vs. CFA samples.

Gene symbol	Genbank accession	GeneName	CFA vs. Normal	WVBF vs. Normal	WVBF vs. CFA
Sult2a5	NM_001184980	Sulfotransferase-family-2A,-dehydroepiandrosterone-(DHEA)-preferring,-member-5	0	NA	NA
A1bg	NM_001081067	Alpha-1-B-glycoprotein	0	NA	NA
Atp6v0d2	NM_175406	ATPase,-H+-transporting,-lysosomal-V0-subunit-D2	0	NA	NA
NA	NA	NA	0	NA	NA
Tc2n	NM_028924	Tandem-C2-domains,-nuclear	0.1	NA	NA
Lrtm1	NM_176920	Leucine-rich-repeats-and-transmembrane-domains-1	0.1	NA	NA
NA	AK079005	NA	0.1	NA	NA
NA	AK079807	NA	0.2	NA	NA
NA	AK012891	NA	0.2	NA	NA
2810002D19Rik	NR_027831	RIKEN-cDNA-2810002D19-gene	0.0	NA	NA
Tbx1	NM_011532	T-box 1	NA	0.1	NA
Prom2	NM_178047	Prominin 2	NA	0.17	NA
Erc2	AK032385	ELKS/RAB6-interacting/CAST family member 2	NA	0.16	NA
S1pr5	NM_053190	Sphingosine-1-phosphate receptor 5	NA	0.17	NA
1700054M17Rik	NR_045919	RIKEN cDNA 1700054M17 gene	NA	0.17	NA
Rgs3	NM_134257	Regulator of G-protein signaling 3	NA	0.18	NA
Rgs3	NM_134257	Regulator of G-protein signaling 3	NA	0.18	NA
Rgs3	NM_134257	Regulator of G-protein signaling 3	NA	0.18	NA
Ctps2	NM_018737	Cytidine 5′-triphosphate synthase 2	NA	0.19	0.19
Egfr	AK049452	Epidermal growth factor receptor	NA	NA	0.14
Kcnk9	XM_006520768	Potassium channel, subfamily K, member 9	NA	NA	0.16
Slc25a29	NM_181328	Solute carrier family 25 (mitochondrial carrier, palmitoylcarnitine transporter), member 29	NA	NA	0.19
Ctps2	NM_018737	Cytidine 5′-triphosphate synthase 2	NA	NA	0.19
Snca	NM_001042451	Synuclein, alpha	NA	NA	0.19
Ahsp	NM_133245	Alpha hemoglobin stabilizing protein	NA	NA	0.19
Hbb-bt	NM_008220	Hemoglobin, beta adult t chain	NA	NA	0.19
Gm6792	NM_001177416	Predicted gene 6792	NA	NA	0.19
1600012P17Rik	BC100356	RIKEN cDNA 1600012P17 gene	NA	NA	0.21
Hbb-bt	NM_008220	Hemoglobin, beta adult t chain	NA	NA	0.21
Hba-a2	M10466	Hemoglobin alpha, adult chain 2	NA	NA	0.138


CFA refers to the chloroform fraction of Aconitum brachypodum Diel; WVBF refers to the water decoction of Veratrilla baillonii Franch. No toxic signs were detected. The numbers of animals in parentheses designate physiological changes. The mice were observed after 5 min, 15 min, 30 min, 1 h, 2 h, and 4 h for signs of toxicity (behavioral changes and mortality).

#### Pathway Analysis

A global view of the pathway modulation in the liver of KM mice is shown as a Venn diagram (Figure 4B). More detail analysis of pathway modulation was described in Figure 8. The pathway analysis indicated that CFA may exert protective effect against some diseases via significantly regulating inflammation/immunity related pathways such as allograft-rejection pathway, graft-versus-host-disease pathway, autoimmune-thyroid-disease pathway, viral myocarditis pathway, type-I-diabetes-mellitus pathway, pathway-in-cancer, tuberculosis pathway and energy metabolism pathway, indicating multiple activities of the folk medicine (Figure 8). Pathway analysis also showed that cell survival related pathways regulated by CFA contained adipocytokine signaling pathway, phagosome pathway, cell-cycle pathway, apoptosis pathway and cell killing-mediated-cytotoxicity signaling pathway. CFA may potentially lead to injury of hepatic cells through regulating above pathways of genes such as tumor necrosis factor receptor superfamily, member 1b, Fas ligand (TNF superfamily, member 6) (FasL), granzyme B (GZMB), wingless-type MMTV ‘integration site family, member 4 (Wnt), p21, tubulin and beta 2B class IIB (TUBB).

WVBF, however, could influence multiple target genes induced by CFA and synergistically affect anticancer, anti-inflammation and anti-diabetes pathways, indicating a positive interaction between these two medicines. Therefore, WVBF could attenuate CFA-induced injury by oppositely regulating the genes in apoptosis pathway and phagosome pathway.

### Effect of WVBF Treated CFA-Induced mRNA Levels of TRAF2 and P21

**FIGURE 9:**
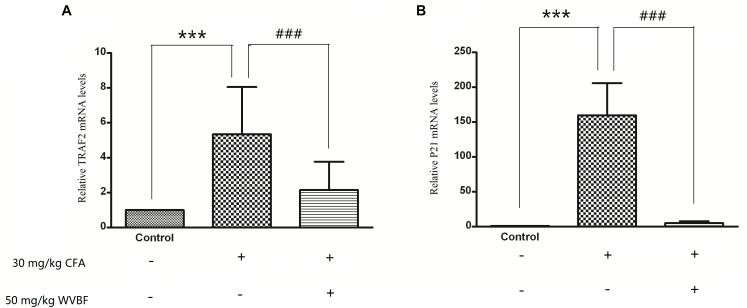
Validation of microarray results by measuring expression of TRAF21 and P21 using real time qRT-PCR. Different bars indicate mean fold change (mean ± SD) derived from duplicate RT-PCR reactions (*n* = 3). ^∗∗∗^*P* < 0.01 vs. Control, ^###^*P* < 0.01 vs. CFA group.

To verify the results of gene profiles, the effect of WVBF treated CFA-induced mRNA levels of TRAF2 and P21 was assessed. As shown in Figure 9, CFA (30 mg/kg) treatment could lead to significant up-regulation of TRAF2 and P21 gene levels. However, WVBF could decrease their gene levels to some degree (*P* < 0.01), indicating anti-apoptotic effect of it. The result was coinciding with microarray data.

## Discussion

As an extract, herbal medicine may contain many bioactive compounds, which may affect multiple pathways in an organism, especially pharmacological doses. To observe the changes of the hepatic gene expression profiles, we decided to use gene array technology. Gene chip refers to the two-dimensional DNA probe array generated through *in situ* synthesis of thousands of DNA probes on the support surface. Gene chip allows quick and simultaneous quantitative expression the of gene levels in a biological sample. It is a powerful tool to identify and characterize changes in genes expression associated with the mechanism of Chinese medicine (Celik and Suzek, 2008). The present study shows WVBF can attenuate CFA-induced liver damage of mice (to see in Figure 9). Furthermore, differentially expressed genes due to the presence of CFA and CFA + WVBF are summarized and discussed as below.

### Genes Involved in the Attenuation of CFA-Induced Liver Toxicity by WVBF

The immune system can trigger T cell antigen receptor (TCR) signaling by activating three pathways: (1) phospholipase Cβ- inositol 1,4,5-triphosphate (PLC-IP_3_), increase intercellular calcium concentration, activate calcineurin (CaN) and its downstream transcription factor nuclear factor of activited T-cells (NF-AT) (Kaysen et al., 2002; Zou et al., 2010); (2) phospholipase Cβ-diacylglycerol-protein kinase C (PLC-DAG-PKC) and its downstream transcription factor Nuclear factor Kb(NF-KB); (3) growth factor receptor bound protein 2-radar absorb support guanosine triphosphate (Grb2-RasGTP), and further promote the cascade reaction of MAPK (mitogen-activated protein kinase), activating transcription factor Fos and Jun (Schmidt et al., 1992; Kang, 2008).

CFA can affect the functions of immune system. CFA treatment probably can inhibit the release of immune molecules such as major histocompatibility complex (MHCI/II) and melanin-concentrating hormone (CD) as well as the information of TCR+CD4/CD8-MHC-Ag, thus destroying the antigen recognition reaction in immune system. In detail, CFA can up-regulate Itga (CD48) and targeting GTPase (Rab31) while down-regulate phospholipase and wingless-type MMTV integration site family (Wnt), and inhibit immune activation pathway PLC-IP3-Ca^2+^-Calcineurin-NFAT and PLC-DAG-PKC-NF-KB. (2) Down-regulate interleukin-1 (IL-1R) for inhibiting the activation of CD4 + T cells; down-regulate interleukin-2 (IL-2R) for inhibiting the amplification of T cell. CFA can inhibit immune system by down-regulating ATPase, which leads to the increase of ATP, activation of NADPH oxidase system, and the promoted release of ROS. As we known, excessive production of ROS will induce functional disorder of mitochondria, thus leading to cell apoptosis/necrosis (Hirata et al., 1995; Garcia-Espinosa et al., 2012). Therefore, CFA is of anti-inflammatory effect at fairly dosage but of cytotoxicity under excessive dosage.

In addition to above toxic pathway, CFA can lead to toxicity by other pathways including:

(i) Activated AMPK and regulated metabolic defect, which will reduce the production of hepatic glycogen and fatty acid, activate H_2_O_2_ and promote oxidative stress in the phagosome pathway, and thus leading to damage of hepatocyte.
(ii) Up-regulated cyclin-dependent kinase inhibitor p21, TRAF-2 as well as activated c-Jun amino-terminal (JNK) gene, which can promote cell apoptosis and necrobiosis. P21 is thought to play a central role in treating inflammatory and cardiovascular disease (Yuen et al., 2011). When p21 is intact, inflammation might divert neuronal progenitors toward astrogliogenesis by inducing p21 (Tsukahara et al., 1993; Dinerman et al., 1994).

However, WVBF could attenuate CFA-induced liver injury by oppositely regulating the genes including:

(i) Up-regulated MHC I/II, microphthalmia-associated transcription factor (MITF), Wnt, myosin and IL-1R, down-regulatedβ-tubulin (TUBB), Rab31 and triggered TCR signaling, activating immune cytokine IL-2, transforming growth factor-β (TGFβ), IL-2 and Fas-ligand [FasL, thus further activating immunoreaction of natural killer cell (NK cells), macrophage, mast cells as well as remove infected or cancer cells]. In addition, WVBF could down-regulate *N*-methyl-D-aspartate receptor (NMDAR) and Synuclein, Alpha (SNCA) and inhibit the cell apoptosis and chronic inflammation induced by NMDAR-Ca^2+^-NOS and SNCA-TNF (tumor necrosis factors)/IL-1 pathways. Moreover, WVBF could up-regulate ATPase, and inhibit oxidative stress, thus reducing the cell damage of liver tissue.
(ii) Up-regulated nAChR (nicotinic acetylcholine receptors) gene of cholinergic synapse pathway. nAChR administrated by ligand binding to regulate ion conduits found in the cellular membrane of excitable and non-excitable cells, respectively. Mitochondrial penetrability transition phase was regulated by ion-independent machinery through α7 nicotinic receptors by activating PI3K/Akt pathway coupling with and the suppression of Src-kinase-dependent signaling pathways (Taglieri et al., 2014), thus suppressing cell apoptosis.

### Potential Synergetic Effect of WVBF and CFA on Targets of Some Diseases

WVBF and CFA have potential synergetic effects on multiple targets of the diseases such as inflammation, cancer and diabetes.

Inflammation and cancer.

CFA could inhibit immune reaction by down regulation of MHCI/II and Toll-like receptors (TLR) genes. The MHCergic system has been investigated to explore the feeding response in relation to energy homeostasis (Ji et al., 2014). TLR activation can induce an instant immune response, which will regulate the relocation of dendritic cells to the infection site (Ko et al., 2015). The two genes MHC and TLR can induce the down regulation of interferon-γ-inducible protein 10 (CXCL10) and v-ATPase genes as well as the up regulation of p21. In addition, CXCL10 and its receptor are increased in many kinds of chronic inflammatory arthritis, especially in rheumatoid arthritis (RA), which can be produced by infected tissue together *in vivo* and *in vitro* (Saito and Nagasaki, 2008; Gergalova et al., 2014). Also, the role of V-ATPase has been associated in several diseases including cancer and antiviral infections (Sugiura et al., 2013). These pathways may interpret the anti-inflammatory and analgesia effect of CFA and the traditional clinical use of the folk medicine.

**FIGURE 10:**
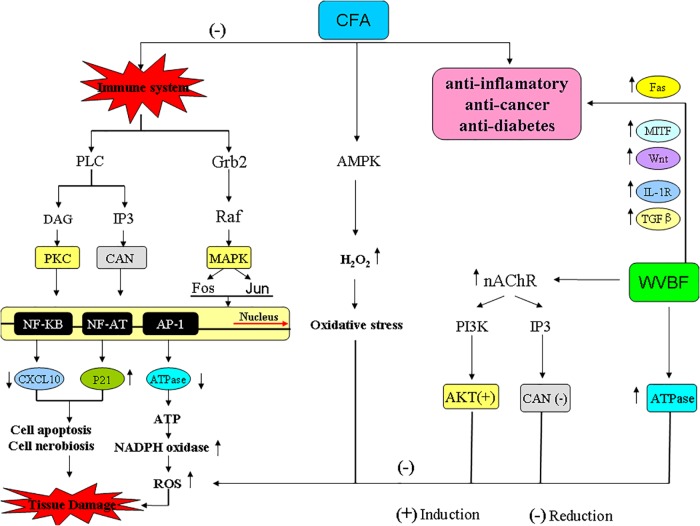
The pathways and genes involved in the interaction of CFA and WVBF.

Moreover, CFA could induce apoptosis and suppress proliferation of cancer cells via inhibiting Wnt and frizzled genes as well as via up regulation of p21, TRAF-2 and integrin alpha 2 ITGA genes in cancer (Figure 10, Lee et al., 2013). The Wnt signaling pathway is essential to regulate cell polarity, cell propagation, and mutation. This pathways is associated many diseases and genetic defects by birth (Klein et al., 2005; Marshansky et al., 2014). The p21 is a has been linked to suppression of tumor, also intricate in many progressions including defense against oxidative stress defense and cell apoptosis (Cabal-Hierro et al., 2014).

Meanwhile, WVBF could influence multiple target genes and synergistically lead to anti-cancer and anti-inflammation function. As for some important genes related to cancer and inflammation, Fas binding ligands restricts injury response in terms of inflammation and allows allograft endurance by removing of Fas-linked lymphocytes through apoptosis (Figure 10, Bienz and Clevers, 2000). The widely distributed cytokine TGF-β is involved in regulating the immune system by inducing T-cell differentiation and endorsing immunosuppression using regulatory T-cells (*T*_regs_) *in vivo* (Wang et al., 2009). Mouse models studies revealed the essential role of TGF-β pathway, as abolition of this pathway is responsible for incurable inflammation (Figure 10, Yang et al., 2014; Plenter et al., 2015). Any mutation in type II TGF-β receptor limited anti proliferative response in many cell lines and tumors (Li et al., 2013; Oh and Li, 2013; Liu and Desai, 2015). A regulatory protein C/EBPβ with peptidase inhibitor is obligatory for controlled expression of genes involved in inflammatory response, is a fundamental inducer of the interleukin (IL)-36α (*Il36A*) gene, which is a effective regulator for accessory and T cells and contribute critically in inflammatory response. WVBF could up-regulate Fas, c-EBPβ, sphingosine kinase-1 (Sphkl), TGF-β, MITF, down-regulated IL-1R, and synergistically lead to anti-inflammatory and anti-cancer effect (Figure 10).

Diabetes

The liver is imperative organ, which govern lipid and glucose condition in the body to maintain homeostasis condition. It is responsible for the production and utilization of glucose in description to a number of nutritional and hormonal and stimuli. Gene expression regulation is the key to monitor glucose and metabolism of lipids in the liver. The Wnt signaling pathways regulate genes through a variety of pathways in tissue specific manner in a complex manner (Kulkarni et al., 1993; Gorelik and Flavell, 2000; Marie et al., 2006). Many studies have revealed that a number of components of the Wnt pathway are complicated in the proliferation of beta cell in pancreas (Gajos-Michniewicz and Czyz, 2016), glucose-induced insulin secretion, normal cholesterol metabolism, glucagon-like peptide-1 and the production of the incretin hormone (Tao et al., 2016).

Our previous study showed that gentiopicroside and sweroside, the main components in WVBF, could suppress Pck1 expression and induce phosphorylation of components in the insulin signaling cascade (Huang et al., 2016). This is the first study to demonstrate that gentiopicroside and sweroside show insulin-mimicking effects on the regulation of Pck1 expression (Huang et al., 2016). The present results showed that CFA could inhibit Granzyme genes in pancreatic islet pathway, thus suppressing the apoptosis of beta cells in islet of pancreas and relieving types 1 and 2 diabetes. However, WVBF could up-regulate MHCI/II and TLR genes, thus promoting the balance of body immune reaction. The two genes MHC and TLR could induce up-regulation of Wnt and MITF gene, and thus handling protective effect against diabetes via promoting the secretion of insulin from islet cell. Further studies are warranted to explore the anti-diabetes potential of the medicinal herb in animals.

In Traditional Chinese Medicine (TCM), toxicity and efficiency are two effects of the drug. The present results of microarray data as well as qPCR verify both attenuation and synergism effects of WVBF on CFA induced mice. This may be a useful guide to compose a formula including aconitum plants. In general, this study gives a systematic explanation of the effect of WVBF on CFA tempted liver tissue. However, other potential mechanism on interaction between the two folk medicines remains to be determined.

## Ethics Statement

Animal protocols were followed according to the National Institutes of Health (NIH) rules (NRC, 2004) and approved by the Ethical Committee in South-Central University of Nationalities (No. yxy20141008).

## Author Contributions

JL, GL, and X-JH designed the experiments. JL, GL, AI, XY, HC, X-JH, AM, and D-GW performed the experiments and analyzed the data. JL, AM, and X-JH wrote the manuscript.

## Conflict of Interest Statement

The authors declare that the research was conducted in the absence of any commercial or financial relationships that could be construed as a potential conflict of interest.

## Footnotes

1 http://www.ebioservice.com

2 http://www.genome.jp/kegg
