# Supplementary material for: Effects of Veratrilla baillonii Extract on Hepatic Gene Expression Profiles in Response to Aconitum brachypodum-Induced Liver Toxicity in Mice
Source: Front Pharmacol. 2019 May 31;10:568. doi: 10.3389/fphar.2019.00568 (PMC6555156; doi:10.3389/fphar.2019.00568)
Supplement: Supplementary file 1 [file Data_Sheet_1.docx]

**Supplementary data**


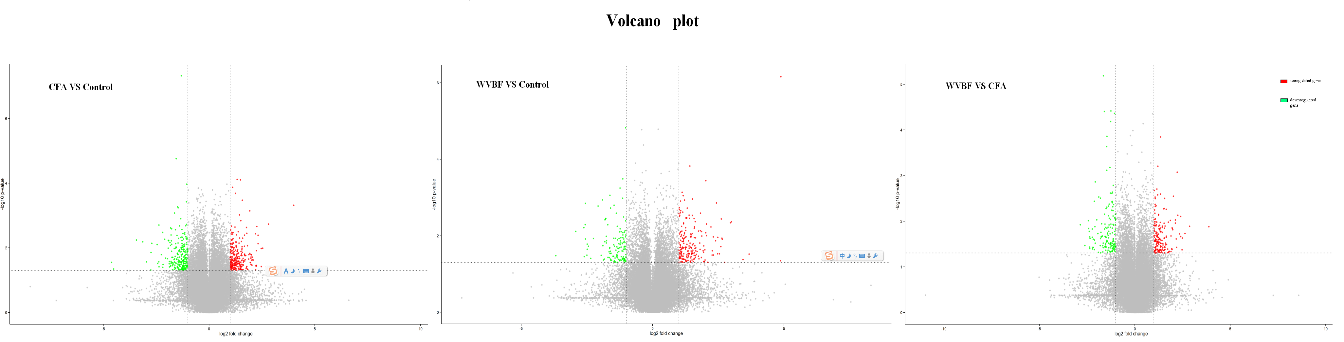


**Supplementary Fig. 1**. Volcano plot, which was drawn based on P-value and fold change checked by t-test, shows the significant difference of sample data between chipset groups. Described by Gene-Spring 11.0, X-axis is for Log2(fold change) and Y-axis is for -Log10 (P-value); X-axis parallel line: *P*＝0.05, Y-axis parallel line: fold change＝2.0; Red Zone：*P*＜0.05 with fold change≥2.0, and Green Zone：*P*＜0.05 with fold change<0.5).


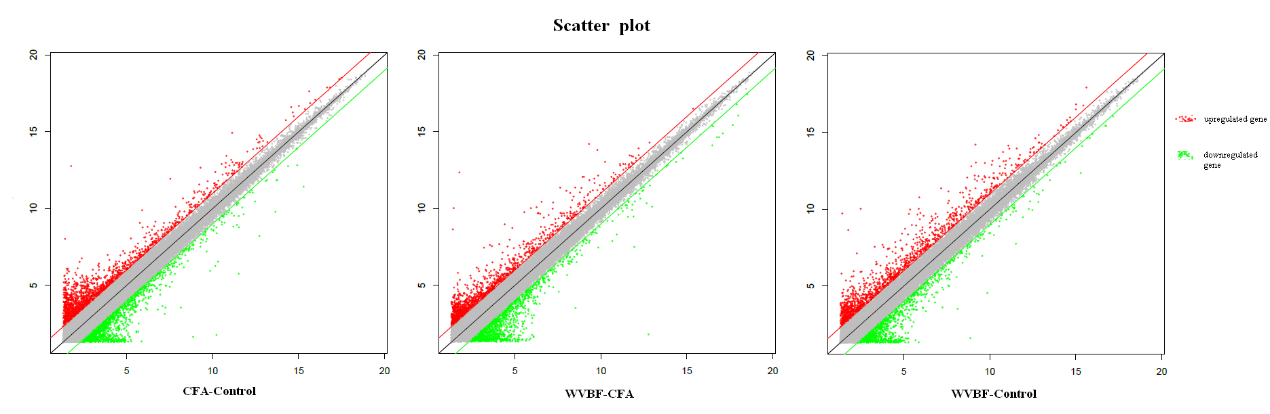


**Supplementary Fig. 2**. Scatter plot of the differentially expressed genes among WVBF, CFA and Control groups. Scatter plot, which was drawn based on fold change checked by t-test, shows the significant difference of sample data between chipset groups. Described by Gene-Spring 11.0, each data point represents a gene hybridization signal on the chip. X-axis and Y-axis are for fold change; If falling in the graphic y = x line (the median line in the figure), then fold change＝2.0; If the median line is on both sides of the 45 line outside the point, then fold change>2.0 or fold change<0.5).

| Gene symbol  CFA vs cont | Gene symbol  WVBF vs cont | Gene symbol WVBF vs CFA | GO (MGI)  CFA | GO (MGI)  WVBF | GO (MGI) WVBF vs CFA |
| --- | --- | --- | --- | --- | --- |
| Zfp945 | Zfp945 | H2-Q1 |  |  | immune system, Response to stimulus |
| Itga2 | Adcy1 | Arhgap20os | cell proliferation, cellular component organization, establishment of localization, immune system process, protein metabolic process, response to stimulus, signaling, system development | carbohydrate derivative metabolism, cell differentiation, cellular component organization, establishment of localization, nucleic acid-templated transcription, response to stimulus, signaling, system development | NA |
| Cml5 | H2-Q1 | Hyls1 | system development | immune system, Response to stimulus | Cellular component organization |
| 4930579F01Rik | Ptgfr | Chrna4 | NA | cell death, cell proliferation, homeostatic process, response to stimulus, signaling | Cellular component organization, establishment of localization, immune system, response to system, singnaling |
| Cspg5 | Ren2 | Vgll3 | cell differentiation, establishment of localization, signaling, system development | protein metabolic process | nucleic acid-templated transcription |
| Ren1 | Ren1 | Krt2 | cell differentiation, establishment of localization, protein metabolic process, response to stimulus, signaling, system development | cell differentiation, establishment of localization, protein metabolic process, response to stimulus, signaling, system development | cell differentiation, cellular component organization, system development |
| Ren2 | Cabyr | A430089I19Rik | protein metabolic process | cell differentiation, cellular component organization, response to stimulus, signaling | NA |
| Ren1 | Pnpla3 | Atp6v0d2 | cell differentiation, establishment of localization, protein metabolic process, response to stimulus, signaling, system development | cellular component organization, homeostatic process, lipid metabolic process, response to stimulus | establishment of localization, homeostatic process |
| Gadd45b | 4930579F01Rik | Gabbr2 | cell death, cell differentiation, protein metabolic process, response to stimulus, signaling | NA | signaling |
| Wfdc12 | Ciart | Tnnt2 | protein metabolic process, response to stimulus | nucleic acid-templated transcription | cell differentiation, cellular component organization, system development, response to stimulus, establishment of localization |

**Supplementary Table 1**: Single gene Annotation for top 10 upregulated genes in CFA, WVBF, WVBF vs CFA

| Gene symbol  CFA vs cont | Gene symbol  CFA vs cont | Gene symbol  CFA vs cont | Gene symbol  CFA vs cont | Gene symbol  CFA vs cont | Gene symbol  CFA vs cont |
| --- | --- | --- | --- | --- | --- |
| Hba-a2 | Tbx1 | Sult2a5 | cell differentiation, homeostatic process, immune system process, response to stimulus, system development | cell death, cell differentiation, cell proliferation, immune system process, nucleic acid-templated transcription, protein metabolic process, response to stimulus, signaling, system development | NA |
| Hbb-bt | Prom2 | A1bg | cellular component organization | cellular component organization,establishment of localization, protein metabolic process | NA |
| 1600012P17Rik | Erc2 | Atp6v0d2 | NA | cellular component organization, establishment of localization, signaling | establishment of localization |
| Gm6792 | S1pr5 | Tc2n | NA | response to stimulus, signaling | NA |
| Hbb-bt | 1700054M17Rik | Lrtm1 | cellular component organization | NA | cellular component organization, system development |
| Ahsp | Rgs3 | 2810002D19Rik | cell differentiation, homeostatic process, immune system process, system development | response to stimulus, signaling | NA |
| Snca | Ctps2 | Dock8 | carbohydrate derivative metabolism, cell death, cellular component organization, establishment of localization,  homeostatic process, immune system process | carbohydrate derivative metabolism | cell death, cell proliferation, immune system process, response to stimulus, signaling |
| Ctps2 | Egfr | Prom2 | carbohydrate derivative metabolism | cell death, cell differentiation, cell proliferation,cellular component analyses, homeostatic process, immune system process, nucleic acid-templated transcription, protein metabolic process, response to stimulus, signaling, system development | cellular component organization, establishment of localization, protein metabolic process |
| Slc25a29 | Cd163 | E130309F12Rik | establishment of localization, response to stimulus | response to stimulus | lipid metabolic process, system development |
| Kcnk9 | Zfp945 | Art5 | establishment of localization | NA | protein metabolic process |

**Supplementary Table 2**: Single gene Annotation for top 10 upregulated genes in CFA, WVBF, WVBF vs CFA
